# 

**Published:** 1889-01

**Authors:** 


### CONTENTS:

Article I.—The Tonsils, (Faucial, Lingual, Pharyngeal and Discrete); their Functions and Relation to Affections of the Throat and Nose. By Scanes Spicer, M. D ..... 385

" II.—Immediate Filling of Root Canals. By Wm. H. Potter, A. B.,D. M. D ..... 397

" III.—The Influence of Maternal Impressions on the Fœtus. By Frederick L. Classen, M. D ..... 402

" IV.—The Transplanting of a Rabbit's Cornea into the Human Eye. By Julian J. Chisolm, M. D ..... 412

" V.—Copper Amalgams ..... 416

" VI.—A Reaction Against Antiseptics ..... 422

Brom-Ethyl as an Anæsthetic ..... 423

### EDITORIAL.

The University of Maryland Dental Department ..... 424

An Impromptu Intero Dental Splint ..... 425

An Incorrect Statement ..... 426

### MONTHLY SUMMARY.

How Shall the Surgeon Disinfect His Instruments? ..... 427

Treatment of Carbuncle ..... 428

The Spread of Antisepsis ..... 429

Virchow on Artificial Deformities ..... 430

Disinfection of Surgical Instruments ..... 431

Oxygen Not so Important in Respiration ..... 431

Verdict of Malpractice in Administration of Chloroform ..... 431

Beef, Milk and Phthisis ..... 432

Opium Smoking ..... 432

Extirpation of the Larynx ..... 432

